# Fetal Growth Restriction Is Associated With Altered Optic Nerve Head Morphology in Term-Born Children and Adolescents

**DOI:** 10.1167/iovs.66.4.35

**Published:** 2025-04-15

**Authors:** Achim Fieß, Sandra Gißler, Stephanie Grabitz, Esther M. Hoffmann, Eva Mildenberger, Timo Uphaus, Marianne Hahn, Norbert Pfeiffer, Alica Hartmann, Alexander K. Schuster

**Affiliations:** 1 Department of Ophthalmology, University Medical Center of the Johannes Gutenberg University Mainz, Mainz, Germany; 2 Division of Neonatology, Department of Pediatrics, University Medical Center of the Johannes Gutenberg University Mainz, Mainz, Germany; 3 Department of Neurology, Focus Program Translational Neuroscience (FTN) and Immunotherapy (FZI), Rhine Main Neuroscience Network (rmn2), University Medical Center of the Johannes Gutenberg University Mainz, Mainz, Germany; 4 Department of Neurology and Focus Program Translational Neuroscience (FTN), Rhine Main Neuroscience Network (rmn2), University Medical Center of the Johannes Gutenberg University Mainz, Mainz, Germany

**Keywords:** fetal growth restriction, retinal nerve fibre layer, minimum rim width, Bruch's membrane opening, cup-to-disc ratio

## Abstract

**Purpose:**

Restricted fetal growth is associated with impaired neurodevelopment in childhood. We examined the effects of fetal growth restriction, fetal overgrowth, and other perinatal parameters on optic nerve head (ONH) morphology in term-born children and adolescents.

**Methods:**

This retrospective cohort study with a prospective ophthalmologic examination included full-term born children aged 4 to 17 years who were grouped according to their birth weight correlated to gestational age (GA). We formed the following groups: severe fetal growth restriction (<3rd birth weight [BW] percentile, group 1), moderate fetal growth restriction (BW percentile 3rd to <10th, group 2), appropriate for gestational age (AGA, 10th–90th BW percentile, group 3, control group), moderate fetal overgrowth (>90th–97th BW percentile, group 4), and severe fetal overgrowth (>97th percentile, group 5). The participants underwent spectral-domain optical coherence tomography and fundus photography to evaluate the peripapillary retinal nerve fiber layer (pRNFL) thickness, minimal rim width (MRW), Bruch's membrane opening (BMO), and vertical cup-to-disc ratio (vCDR), focusing on their relationship to perinatal factors like nutritional status, GA, maternal smoking, and maternal breastfeeding. The relationships between the ONH parameters and perinatal factors were adjusted for variables such as age, sex, and axial length.

**Results:**

This study included 732 eyes of 375 participants (mean age of 11.4 ± 3.71 years, 193 female subjects). Multivariable regression analyses showed an association between a thinner global pRNFL thickness in the participants with severe fetal growth restriction (B = −4.95; 95% confidence interval [CI], −9.43 to −0.47 µm; *P* = 0.03) compared to the reference AGA group. Furthermore, an association with a thinner MRW was found in the children born with moderate fetal growth restriction (B = −32.46; 95% CI, −51.52 to −13.40 µm; p < 0.001). BW percentile was associated with median vCDR (B = −0.001; 95% CI, −0.002 to 0.00; *P* = 0.02). No consistent association was observed between altered fetal growth and BMO.

**Conclusions:**

Severe fetal growth restriction appears to affect the optic nerve head in term-born children and adolescents, suggesting a possible reduction in neuronal reserve, and may indicate a potentially elevated risk of abnormal neurodevelopment.

Restricted fetal growth is a significant risk factor for both short- and long-term childhood morbidity, with substantial effects on the central nervous system.^1^ It has been linked to a range of developmental disorders, including deficits in neurologic and intellectual outcomes, compared to children born appropriate for gestational age (AGA).^2^^–^^5^ Fetal growth restriction at birth is classified as severe (severely small for gestational age [SGA]) when birth weight is below the 3rd percentile and moderate when birth weight falls between the 3rd and less than the 10th percentile relative to gestational age.^6^ Fetal overgrowth at birth is classified as severe (large for gestational age [LGA]) when birth weight is above the 97th percentile and moderate when birth weight falls between the 90th and less than the 97th percentile relative to gestational age.

Worldwide, approximately 30 million infants are born with fetal growth restriction each year,^6^ and they may have a reduced total brain volume and decreased cortical gray matter.^2^^,^^7^ Research also suggests a link between brain structure and the peripapillary retinal nerve fiber layer (pRNFL) measurable by optical coherence tomography (OCT).^8^ Since the optic nerve head is part of the central nervous system, pRNFL measurements provide a noninvasive assessment of neuronal tissue and detect potential central nervous system degeneration as observed in conditions like multiple sclerosis^9^ or Alzheimer disease.^10^ Thinning of the pRNFL is particularly relevant for ocular diseases, as it is associated with glaucoma, the leading cause of irreversible blindness worldwide.^11^ A higher birth weight percentile, however, may therefore serve as a protective factor regarding pRNFL thinning and has been reported in participants with high birth weight in a population-based study.^12^ However, this study was not able to adjust for gestational age as this parameter was not available.^12^^,^^13^

Previous studies have primarily focused on the degenerative relationship between prematurity and pRNFL thickness,^14^^–^^19^ as well as the impact of low birth weight on pRNFL.^12^ However, the effects frequently cannot be separated from each other because of the close correlation between preterm birth and low birth weight as most studies did not exclude preterm children from their analyses and did not take birth weight percentiles into account. One study in children found no significant link between birth weight and optic disc morphology in individuals born full-term.^20^ Another study in adults reported no association between low birth weight and the vertical cup-to-disc ratio (vCDR) and pRNFL,^21^ while a recent report detected a lower pRNFL^22^ and increased vCDR in participants born with fetal growth restriction.^23^ Consequently, the onset and persistence of the effects of altered fetal growth remain unclear, posing significant challenges to understanding their long-term implications. The variability in outcomes may be influenced by a range of factors, including the degree of growth restriction or fetal overgrowth, the timing of intervention, individual biological differences, and the complexity of interactions between prenatal conditions and postnatal environments, emphasizing the need for further research to elucidate these critical aspects.

This study was designed to examine the impact of altered fetal growth, consisting of fetal growth restriction and fetal overgrowth, on optic nerve head (ONH) morphology, including a comprehensive analysis of the OCT parameters pRNFL thickness, minimum rim width (MRW), size of the area of Bruch's membrane opening (BMO), and several morphologic features of the optic disc such as the vCDR in a large cohort. The unique focus on term-born children distinguishes the effects of altered fetal growth from other perinatal parameters, avoiding the confounding effects of preterm birth commonly present in prior research. By including the rarely analyzed OCT parameters MRW and BMO, this study offers new insights into the impact of altered fetal growth on both neurodevelopmental outcomes and ocular development.

## Materials and Methods

### Study Population and Categorization

This study is a retrospective single-center cohort study with a prospective ophthalmologic examination conducted at the University Medical Center of the Johannes Gutenberg-University Mainz (UMCM) in Germany. It involved a thorough ophthalmologic examination and a detailed questionnaire completed by individuals born full-term (≥37 weeks) between 2003 and 2018. The participants, now aged 4 to 17 years, were recruited from birth records stored at the UMCM. For every month, we invited eight (four male/four female) randomly selected full-term individuals born in the respective month with a birth weight (BW) between the 10th and 90th percentile (AGA), every child born with severe fetal growth restriction, every second child born with moderate fetal growth restriction, every second child born with moderate fetal overgrowth, and every child born with severe fetal overgrowth. The recruited participants were categorized into the five groups and matched by age: group 1, 30 individuals born with severe fetal growth restriction; group 2, 30 individuals born with moderate fetal growth restriction; group 3, 260 individuals born AGA (control group); group 4, 30 individuals born with moderate fetal overgrowth; and group 5, 30 individuals born with severe fetal overgrowth (see flowchart in Supplementary Fig. S1; in figures and tables, the abbreviations SGA and LGA will be used for fetal growth restriction and fetal overgrowth, respectively). We also reviewed the participants’ and their mothers’ data from maternal peri- and postnatal medical files. Each participant underwent a prospective extensive ophthalmologic examination.

Inclusion criteria consisted of term birth between 2003 and 2018 at the UMCM with additional age-matching between the five groups and balancing participant numbers between the groups, while exclusion criteria consisted of intraocular pressure above 25 mm Hg in the examination and/or glaucomatous atrophy in optic disc photographs as well as unsuccessful optic disc and pRNFL measurements.

We obtained written informed consent from all participants and their legal guardians before study entry. This study complied with Good Clinical Practice, Good Epidemiological Practice, and the ethical principles of the Declaration of Helsinki. The study protocol and study documents were approved by the local ethics committee of the Medical Chamber of Rhineland-Palatinate, Germany (reference no. 2021-15830; original vote: May 5, 2021, latest update: January 19, 2022).

### Assessment of Pre-, Peri-, and Postnatal Medical History

We examined the medical birth records at the University Medical Center Mainz to gather data on gestational age (GA); birth weight; placental insufficiency; hemolysis, elevated liver enzyme levels, and low platelet levels (HELLP); breastfeeding; maternal smoking; gestational diabetes; and preeclampsia. Birth weight percentiles were calculated based on the method established by Voigt et al.^24^ Preeclampsia and HELLP syndrome were defined based on the Guideline Program of the German Society for Gynecology and Obstetrics, a definition consistently used for several years.^25^ Gestational diabetes was diagnosed using the two-step screening test established at the UMCM in 2012, following the S3 Guideline Gestational Diabetes Mellitus.^26^ The definition of chronic placental insufficiency was based on Doppler sonography, amniotic fluid assessment, and exclusion of differential diagnoses (e.g., TORCH infections, chromosomal abnormalities), as histopathologic examination of the placenta was not always available. While placental insufficiency is widely discussed, its definition remains fluid and subject to ongoing debate among scholars.^27^

### Ophthalmologic Examination

The comprehensive ophthalmologic assessment involved testing of distant corrected visual acuity using an ARK 1s (NIDEK; Oculus, Wetzlar, Germany) and measuring axial length with a LenStar LS900 (Haag-Streit, Bern, Switzerland). We measured intraocular pressure using a noncontact tonometer (Nidek NT-2000; Nidek Co., Gamagori, Japan). Visual acuity was converted from decimal to logMAR following established medical guidelines.^28^

### Optical Coherence Tomography

We performed spectral-domain OCT of the peripapillary region using a 12° diameter scan centered on the optic disc, with eye-tracking and standard settings of 7.7 mm corneal curvature and ametropia of 0 diopters (D). Automated layer segmentation was conducted using the Heidelberg Eye Explorer Software (HEYEX, version 6.13.3.0) to calculate global and sectorial pRNFL thickness (global, superonasal, nasal, inferonasal, inferotemporal, temporal, and superotemporal) (see Supplementary Fig. S3). Adjustments for ocular magnification were made based on corneal curvature and spherical equivalent. Additionally, 24 high-resolution ONH radial scans and 3 circle scans were obtained using the ONH radial circle scan from the Glaucoma Module Premier Edition software (version 6.10; Heidelberg Engineering, Heidelberg, Germany). Bruch's membrane opening minimum rim width (BMO-MRW) was defined as the shortest distance from the BMO to the internal limiting membrane and was automatically calculated globally and by sector (see Supplementary Fig. S2). Image quality was evaluated for centering, focus, and accurate segmentation, and images were manually corrected or excluded if segmentation errors occurred or the quality was poor (<15 db).

### Fundus Photography and Optic Disc Measurements

Fundus photography of the ONH was performed in a darkened room with dilated pupils using a Visucam PRO NM (Carl Zeiss Meditec AG, Jena, Germany) system capturing 30° and 45° images, starting with the right eye. Regular quality control was maintained. An experienced, blinded investigator manually measured optic disc parameters using ImageJ software (version 1.53a; National Institutes of Health, Bethesda, MD, USA). The parameters assessed included vertical disc and cup lengths, vCDR, longer and shorter disc diameters, optic disc area, torsion angle, and the presence of disc tilt or torsion. All measurements were adjusted for ocular magnification according to the method by Garway-Heath et al.^29^

### Covariates

The study covariates included age (years), sex (female), axial length (in mm), optic disc area (in mm²), GA (in weeks), BW, birth weight percentile group (1–5, with group 3 as the reference), and whether the mother experienced preeclampsia, had placental insufficiency, smoked during pregnancy, experienced HELLP, or breastfed.

### Statistical Analysis

The main outcome measures were global peripapillary RNFL thickness, the global MRW, the BMO area, and the vCDR. Descriptive statistics were stratified by groups, and absolute and relative frequencies were calculated for dichotomous parameters, with the mean and standard deviation calculated for approximately normally distributed variables, otherwise median and interquartile range. One-way ANOVA was used to compare continuous and approximately normally distributed variables and the χ^2^ test for dichotomous variables. The Kruskal–Wallis test was used to compare continuous variables with nonnormal distribution. Linear regression with generalized estimating equations was conducted for RNFL thickness, global MRW, and BMO area analysis in a complete case-analysis set to account for intereye correlations within each participant.

Associations were analyzed with a linear quantile mixed model with the eyes set as a random factor to account for intereye correlation due to skewness in the vCDR distribution. The median (τ = 0.5) was used to calculate the estimator.

Crude univariable analyses were performed for all outcome variables regarding the influence of GA (weeks), birth weight percentile category (groups 1–5), maternal smoking during pregnancy (yes), and breastfeeding (yes). The model was adjusted for age (years), sex (female), axial length (mm), and optic disc area (mm^2^) for vCDR. Placental insufficiency, preeclampsia, and HELLP were removed from the analysis due to insufficient participant numbers. We additionally conducted an analysis with the inclusion of all other potential confounders (gestational diabetes, maternal smoking during pregnancy, and breastfeeding) in a multivariable model due to potentially hidden confounding effects.^30^ In another sensitivity analysis, we excluded all participants with an anamnestic history of high IOP, glaucoma, and cerebral bleeding, while we had no cases of cerebral ischemia, neurodegenerative diseases, and cerebral paresis.

As this is an explorative study, a significance level was not defined, and no adjustment for multiple testing was conducted; thus, *P* values are reported only for descriptive purposes and should be interpreted with caution.^31^ Calculations were performed using R (R Foundation for Statistical Computing, Vienna, Austria, R version 4.1.2 (2021-11-01)).

## Results

### Participant Characteristics

In total, 732 eyes of 375 participants (mean age = 11.4 ± 3.71 years, 193 female subjects) were included in the study with successful optic disc and pRNFL measurements. All subjects had normal IOP (≤25 mm Hg), and optic disc photographs were evaluated by a glaucoma specialist, with no glaucomatous optic atrophy detected. Overall, 502 eyes of 257 participants born AGA (group 3, control group), 57 eyes of 29 participants born with extreme fetal growth restriction (group 1), 58 eyes of 30 participants born with moderate fetal growth restriction (group 3), 57 eyes of 29 participants born with moderate fetal overgrowth (group 4), and 58 eyes of 30 participants born with extreme fetal overgrowth (group 5) were assessed, and a summary of the group characteristics is presented in Table 1.

**Table 1.** Characteristics of the Study Sample (*n* = 375), Stratified by Study Groups

Characteristic	Group 1: <3rd Birth Weight Percentile	Group 2: 3rd to <10th Birth Weight Percentile	Group 3: 10th to 90th Birth Weight Percentile	Group 4: >90th to 97th Birth Weight Percentile	Group 5: >97th Birth Weight Percentile
Participants (*n*)/eyes (*n*)	29/57	30/58	257/502	29/57	30/58
Sex (female), *n* (%)	16 (55.2)	15 (50.0)	137 (53.3)	10 (34.5)	15 (50.0)
Age, mean (SD), y	11.6 (4.0)	10.1 (4.0)	11.3 (3.6)	10.6 (3.8)	10.5 (4.0)
Age range, minimum–maximum, y	4–17	4–17	4–17	4–17	4–17
Gestational age, mean (SD), wk	38.5 (1.2)	38.9 (1.6)	39.0 (1.3)	39.1 (1.0)	39.3 (1.3)
Birthweight, mean (SD), kg	2.32 (0.29)	2.68 (0.27)	3.37 (0.40)	4.18 (0.18)	4.53 (0.28)
Birthweight <1500 g (yes), *n* (%)	1 (3.4)	0 (0.0)	0 (0.0)	0 (0.0)	0 (0.0)
Birthweight <1000 g (yes), *n* (%)	0 (0.0)	0 (0.0)	0 (0.0)	0 (0.0)	0 (0.0)
Birthweight percentile, mean (SD)	1.34 (0.48)	5.17 (1.98)	44.71 (24.74)	94.17 (2.14)	99.04 (0.76)
Preeclampsia (yes), *n* (%)	0 (0.0)	1 (3.3)	4 (1.6)	0 (0.0)	1 (3.3)
Placental insufficiency (yes), *n* (%)	2 (6.9)	0 (0.0)	0 (0.0)	0 (0.0)	0 (0.0)
Gestational diabetes, *n* (%)	3 (10.3)	4 (13.3)	32 (12.5)	4 (13.8)	3 (10.0)
Maternal smoking during pregnancy (yes), *n* (%)	3 (10.3)	0 (0.0)	12 (4.7)	1 (3.4)	1 (3.3)
Breastfeeding (yes), *n* (%)	22 (75.9)	25 (83.3)	222 (86.4)	24 (82.8)	26 (86.7)
Ocular parameters					
Visual acuity (logMAR) (OD), median [IQR]	0.00	0.00	0.00	0.00	0.00
	[0.00, 0.10]	[0.00, 0.18]	[0.00, 0.10]	[0.00, 0.00]	[0.00, 0.10]
Visual acuity (logMAR) (OS), median [IQR]	0.00	0.08	0.00	0.00	0.00
	[0.00, 0.10]	[0.00, 0.20]	[0.00, 0.10]	[0.00, 0.20]	[0.00, 0.10]
Spherical equivalent (D) (OD), mean (SD)	0.10 (1.79)	−0.01 (3.26)	0.26 (1.42)	0.59 (1.45)	0.16 (1.44)
Spherical equivalent (D) (OS), mean (SD)	0.14 (1.89)	0.09 (2.97)	0.24 (1.55)	0.52 (1.64)	0.24 (1.67)
Intraocular pressure (mm Hg) (OD), mean (SD)	16.61 (2.94)	17.17 (2.80)	17.00 (3.08)	15.65 (2.52)	16.50 (3.21)
Intraocular pressure (mm Hg) (OS), mean (SD)	16.20 (3.34)	16.41 (2.65)	16.38 (2.91)	15.27 (2.48)	16.53 (3.00)

IQR, interquartile range; OD, right eye; OS, left eye; SD, standard deviation; wk, weeks; y, years.

### Descriptive Sectorial Papillary Measures

Descriptive papillary measurements for pRNFL, BMO area, and MRW are displayed in Table 2, showing significant differences in the global pRNFL and nasal MRW between means in at least two of the groups (*P* ≤ 0.005 for the RNFL and *P* ≤ 0.05 for the MRW). Furthermore, the mean of the pRNFL in the children born with severe fetal overgrowth was significantly smaller (*P* ≤ 0.01), and the pRNFL of the children born with moderate fetal overgrowth was significantly larger (*P* ≤ 0.01) than the pRNFL of the control group. The MRW was significantly smaller in the children born with moderate fetal growth restriction compared to the ones born AGA (*P* ≤ 0.001). The graphical display of measurement distributions in the study groups is shown in the Figure. Regarding the papillary measurements in the fundus photographs, there was only a slightly significant difference in the groups in the tort of the disc, but this was only in the right eyes, and there were no significant differences in the vCDR in both eyes (Table 2).

**Table 2.** Summary of the Optic Nerve Head Parameters According to Study Group

Characteristic	Group 1: <3rd Birth Weight Percentile	Group 2: 3rd to <10th Birth Weight Percentile	Group 3: 10th to 90th Birth Weight Percentile	Group 4: >90th to 97th Birth Weight Percentile	Group 5: >97th Birth Weight Percentile	*P* Value
Eyes (OD/OS), *n*	28/26	26/24	224/218	27/25	26/25	
RNFL (µm) (12° circle scan)						
Global	98.39 (10.75)^**^	103.34 (10.30)	102.55 (9.93)	105.58 (7.38)^**^	104.37 (8.03)	0.002
Superotemporal	136.14 (21.06)	145.34 (19.98)	148.26 (20.38)	151.74 (25.16)	143.83 (19.26)	<0.001
Temporal	70.94 (14.96)	74.32 (13.61)	73.42 (13.06)	77.04 (14.22)	76.28 (16.74)	0.12
Inferotemporal	140.19 (20.70)	145.75 (20.70)	145.81 (18.05)	152.64 (18.19)	151.83 (20.50)	0.003
Inferonasal	110.22 (27.30)	119.05 (28.09)	114.73 (25.52)	116.15 (23.40)	117.07 (29.60)	0.5
Nasal	71.31 (16.88)	74.43 (19.24)	72.44 (16.10)	75.99 (15.37)	76.14 (17.06)	0.3
Superonasal	116.27 (19.79)	119.34 (20.33)	120.08 (22.72)	118.39 (21.95)	117.71 (20.86)	0.8
BMO area (mm^2^)	2.05 (0.40)	2.04 (0.37)	2.03 (0.43)	2.15 (0.60)	2.00 (0.43)	0.72
MRW (µm)						
Global	382.62 (72.51)	351.99 (36.50)^***^	379.92 (58.22)	397.84 (87.90)	388.19 (74.70)	0.06
Superotemporal	361.27 (71.47)	344.09 (45.48)	363.01 (67.13)	381.10 (87.63)	371.68 (68.68)	0.31
Temporal	276.41 (72.59)	261.94 (34.83)	274.78 (53.21)	290.59 (61.83)	291.80 (53.78)	0.17
Inferotemporal	418.94 (86.90)	393.10 (42.82)	417.14 (68.14)	431.43 (75.92)	425.31 (64.23)	0.25
Inferonasal	471.38 (76.02)	426.48 (54.34)	468.12 (70.02)	468.49 (106.00)	472.63 (93.63)	0.05
Nasal	413.21 (87.88)	368.82 (49.81)	410.37 (69.43)	428.86 (120.94)	415.61 (102.93)	0.03
Superonasal	433.72 (80.98)	400.45 (54.44)	424.25 (78.55)	466.29 (115.42)	424.66 (92.33)	0.05
Optic disc characteristics						
Eyes OD (*n*)	26	28	240	27	28	
Vertical disc length (mm)	1.70 (0.18)	1.78 (0.19)	1.73 (0.18)	1.80 (0.23)	1.72 (0.24)	0.3
Vertical cup length (mm)	0.51 (0.25)	0.59 (0.23)	0.48 (0.26)	0.49 (0.36)	0.44 (0.33)	0.3
vCDR, median [IQR]	0.30 [0.21, 0.38]	0.31 [0.25, 0.41]	0.26 [0.15, 0.38]	0.19 [0.14, 0.35]	0.18 [0.13, 0.38]	0.07
Longer disc diameter (mm)	1.71 (0.16)	1.71 (0.15)	1.73 (0.18)	1.77 (0.23)	1.72 (0.22)	0.7
Shorter disc diameter (mm)	1.49 (0.20)	1.53 (0.17)	1.54 (0.16)	1.59 (0.20)	1.54 (0.20)	0.5
Optic disc area (mm^2^)	2.03 (0.44)	2.07 (0.39)	2.12 (0.43)	2.24 (0.58)	2.11 (0.54)	0.6
Torsion angle (absolute), deg, median [IQR]	13.00 [7.50, 21.00]	14.00 [7.75, 16.00]	17.00 [10.25, 27.50]	16.00 [10.50, 24.00]	21.00 [10.00, 28.00]	0.03
Tilted (yes), *n* (%)	4 (16.7)	0 (0.0)	10 (4.8)	0 (0.0)	2 (7.4)	0.06
Torted (yes), *n* (%)	9 (37.5)	7 (29.2)	121 (57.6)	12 (52.2)	14 (51.9)	0.05
Eyes OS (*n*)	26	28	221	23	24	26
Vertical disc length (mm)	1.70 (0.19)	1.76 (0.16)	1.73 (0.17)	1.76 (0.22)	1.75 (0.22)	0.6
Vertical cup length (mm)	0.50 (0.28)	0.55 (0.21)	0.47 (0.26)	0.46 (0.34)	0.40 (0.29)	0.4
vCDR, median [IQR]	0.27 [0.16, 0.38]	0.30 [0.23, 0.37]	0.26 [0.16, 0.37]	0.18 [0.14, 0.35]	0.17 [0.12, 0.31]	0.10
Longer disc diameter (mm)	1.70 (0.19)	1.76 (0.17)	1.74 (0.16)	1.79 (0.22)	1.76 (0.18)	0.5
Shorter disc diameter (mm)	1.51 (0.19)	1.58 (0.18)	1.55 (0.15)	1.61 (0.17)	1.57 (0.17)	0.2
Optic disc area (mm^2^)	2.04 (0.46)	2.21 (0.44)	2.14 (0.40)	2.28 (0.49)	2.20 (0.46)	0.3
Torsion angle (absolute),	18.00	21.50	21.00	21.00	21.50	0.7
deg, median [IQR]	[11.25, 25.75]	[10.50, 28.00]	[13.50, 31.00]	[12.00, 26.00]	[15.00, 34.25]	
Tilted (yes), *n* (%)	2 (8.7)	1 (3.8)	7 (3.6)	2 (9.5)	1 (4.8)	0.6
Torted (yes), *n* (%)	12 (54.5)	15 (62.5)	118 (67.4)	11 (57.9)	12 (66.7)	0.7

Values are presented as mean (SD) unless otherwise indicated. BMO area and MRW values are displayed as mean (SD) of OD + OS.

** *P* ≤ 0.01 compared to controls.

*** *P* ≤ 0.001 compared to controls.

**Figure.**
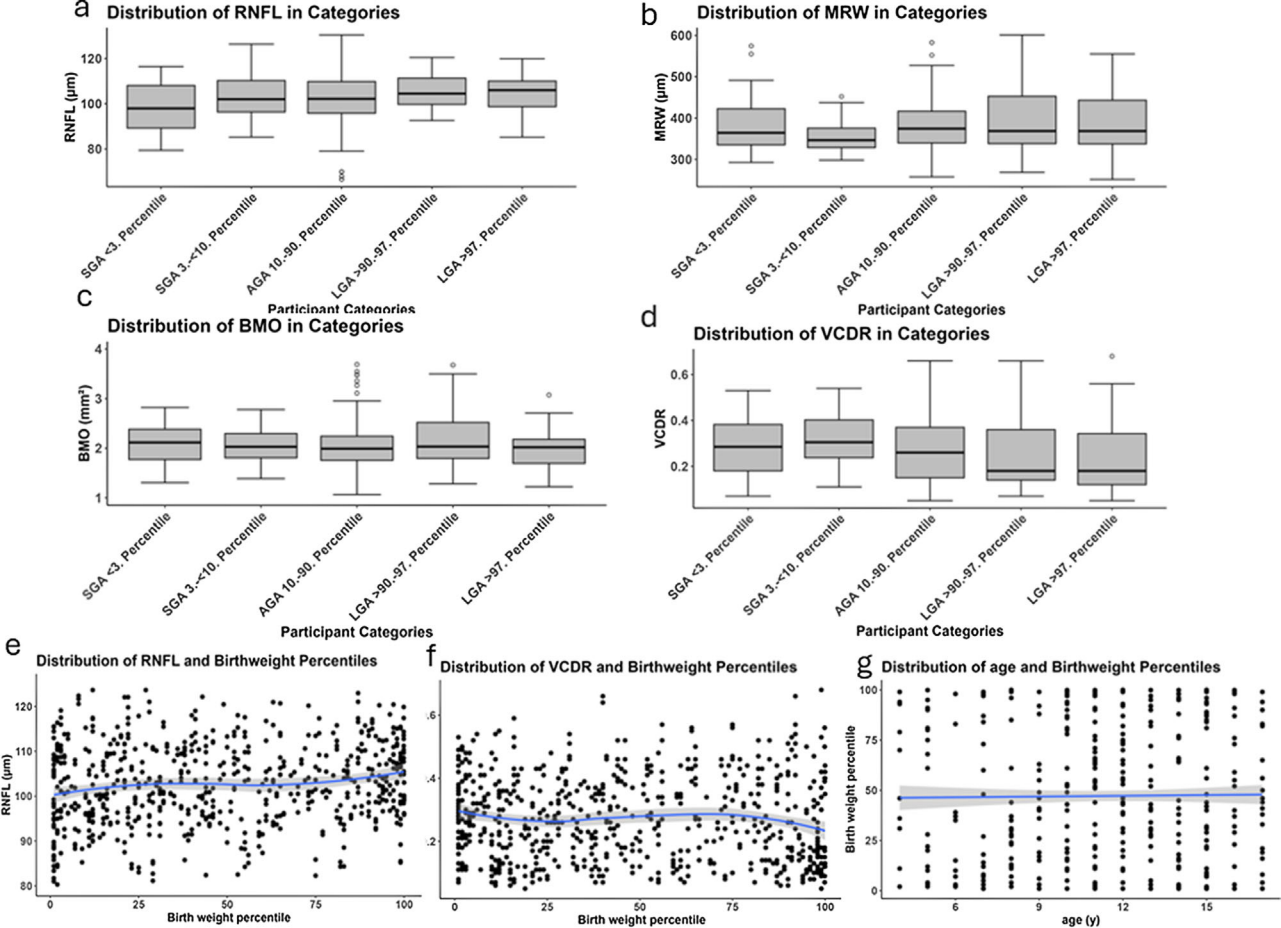
Descriptive group-stratified distribution of (**a**) global retinal nerve fiber layer, (**b**) global minimum rim width, (**c**) Bruch's membrane opening area, (**d**) vertical cup-to-disc ratio in both eyes as well as scatterplots of (**e**) global retinal nerve fiber layer, (**f**) vertical cup-to-disc ratio, (**g**) age versus birth weight percentile.

### Regression Analyses

The adjusted linear regression analyses revealed that only severe fetal growth restriction was significantly associated with a globally thinner pRNFL (β = −5.30, *P* = 0.02), the global MRW was significantly associated with breastfeeding (β = 23.3, *P* = 0.01) and moderate fetal growth restriction (β = −31.99, *P* < 0.001) but not with severe fetal growth restriction, and BMO area was not associated with any of the factors assessed (Table 3). There were no significant associations with fetal overgrowth and the pRNFL, MRW, or BMO. The inclusion of potential confounders in a multivariable model regarding the pRNFL did not show any extreme changes in the estimators and significance, showing the robustness of the associations regarding the additional variables (pRNFL and children born with severe fetal growth restriction: β = −4.95, *P* = 0.03; MRW and children born with moderate fetal growth restriction: β = −32.46, *P* ≤ 0.001), indicating that children born with extreme fetal growth restriction have a mean decrease in pRNFL thickness of about 4.95 µm compared to children born AGA and children born with moderate fetal growth restriction have a mean decrease in MRW of 32.46 µm compared to children born AGA. The significant negative association regarding global MRW and moderate fetal growth restriction but not severe fetal growth restriction, however, shows inconclusive results and may be attributed to unmeasured confounders.

**Table 3.** Association Analyses of Global Peripapillary Retinal Nerve Fiber Layer and Global Minimum Rim Width

	Unadjusted		Adjusted		Multivariable	
Factor	Β (95% CI)	*P*	Β (95% CI)	*P*	Β (95% CI)	*P*
RNFL						
Gestational age	0.21 (−0.72 to 1.16)	0.66	0.22 (−0.71 to 1.15)	0.64	−0.08 (−1.02 to 0.87)	0.87
Birth weight percentile						
<3rd	−5.29 (−9.75 to −0.83)	0.02	−5.30 (−9.67 to −0.94)	0.02	−4.95 (−9.43 to −0.47)	0.03
3rd to <10th	1.25 (−3.49 to 5.99)	0.60	1.49 (−3.08 to 6.06)	0.52	1.31 (−3.22 to 5.83)	0.57
10th to 90th	Reference		Reference		Reference	
>90th to 97th	2.43 (−0.56 to 5.43)	0.11	2.79 (−0.17 to 5.76)	0.07	2.89 (−0.15 to 5.93)	0.06
>97th	3.04 (−0.52 to 6.59)	0.09	3.24 (−0.10 to 6.59)	0.06	3.25 (−0.21 to 6.71)	0.07
Gestational diabetes (yes)	0.95 (−2.92 to 4.82)	0.63	1.0 (−2.84 to 4.84)	0.61	0.85 (−3 to 4.7)	0.67
Smoking during pregnancy (yes)	−2.42 (−6.57 to 1.73)	0.25	−2.84 (−7.09 to 1.40)	0.19	−1.86 (−6.07 to 2.34)	0.39
Breastfeeding (yes)	2.01 (−1.01 to 5.02)	0.19	1.94 (−1.10 to 4.98)	0.21	1.62 (−1.34 to 4.57)	0.28
MRW						
Gestational age	−2.73 (−8.89 to 3.43)	0.39	−2.37 (−8.45 to 3.71)	0.45	−6.01 (−12.51 to 0.49)	0.07
Birth weight percentile						
<3rd	−3.55 (−37.77 to 30.68)	0.84	−9.37 (−41.52 to 22.79)	0.57	−7.61 (−41.21 to 25.99)	0.66
3rd to <10th	−27.01 (−47.03 to −6.98)	0.01	−31.99 (−51.55 to −12.42)	<0.001	−32.46 (−51.52 to −13.4)	<0.001
10th to 90th	Reference		Reference		Reference	
>90th to 97th	28.12 (−16.44 to 72.68)	0.22	30.00 (−15.54 to 75.55)	0.2	30.56 (−13.19 to 74.31)	0.17
>97th	13.17 (−28.28 to 54.63)	0.53	12.89 (−27.94 to 53.72)	0.54	18.27 (−20.84 to 57.37)	0.36
Gestational diabetes (yes)	−20.03 (−41.69 to 1.63)	0.07	−20.33 (−41.94 to 1.28)	0.07	−19.04 (−40.86 to 2.79)	0.09
Smoking during pregnancy (yes)	6.35 (−28.77 to 41.46)	0.72	2.33 (−33.68 to 38.35)	0.9	0.28 (−30.51 to 31.07)	0.99
Breastfeeding (yes)	20.09 (1.01 to 39.18)	0.04	23.3 (5.75 to 40.85)	0.01	27.12 (8.19 to 46.06)	<0.001

Linear regression analysis using generalized estimating equations to control for correlations between OD and OS. Unadjusted—crude model; adjusted—adjusted for age (years), sex (female), axial length (mm).

### Sensitivity Analyses

In the sensitivity analysis with the exclusion of participants with an anamnestic history of high IOP, glaucoma, and cerebral bleeding (there were no reports of cerebral ischemia, neurodegenerative diseases, and cerebral paresis), the estimator for the association between pRNFL and children born with severe fetal growth restriction remained of similar size and was also still significant (β = −5.31, *P* = 0.02). For the MRW, the association with children born with moderate fetal growth restriction also remained the same in the sensitivity analysis (β = −33.28, *P* < 0.001) (see Supplementary Table S1).

### Quantile Mixed-Model Association Analyses of vCDR

The linear quantile mixed-model regression analyses of vCDR with the median as estimator revealed an association with birth weight percentile (β = −0.0007; 95% confidence interval [CI], −0.001 to 0.0; *P* = 0.01) in the adjusted analyses (Table 4). In multivariable analyses, the association with BW percentile (β = −0.001; 95% CI, −0.002 to 0.0; *P* = 0.02) remained, indicating that for around an increase in about 10 BW percentiles, median vCDR decreases by about 0.01 units.

**Table 4:** Linear Quantile Mixed Model Association Analyses of Vertical Cup-to-Disc Ratio

	Unadjusted		Adjusted		Multivariable	
Factor	β (95% CI)	*P*	β (95% CI)	*P*	β (95% CI)	*P*
vCDR						
Gestational age	−0.002 (−0.02 to 0.01)	0.78	−0.00 (−0.01 to 0.01)	0.87	0.003 (−0.01 to 0.02)	0.65
Birth weight percentile	−0.0006 (−0.001 to 0.00)	0.09	−0.0007 (−0.001 to 0.00)	0.01	−0.001 (−0.002 to 0.00)	0.02
Smoking during pregnancy (yes)	0.02 (−0.06 to 0.11)	0.57	−0.004 (−0.09 to 0.08)	0.92	−0.009 (−0.11 to 0.09)	0.85
Breastfeeding (yes)	0.005 (−0.04 to 0.05)	0.81	0.02 (−0.02 to 0.05)	0.33	0.02 (−0.02 to 0.06)	0.40
Gestational diabetes (yes)	0.08 (−0.001 to 0.16)	0.05	0.04 (−0.005 to 0.08)	0.08	0.04 (−0.007 to 0.09)	0.09

Unadjusted—crude model; adjusted—adjusted for age (years), sex (female), axial length (mm), and optic disc area (mm^2^).

## Discussion

To our knowledge, this is the first study to comprehensively evaluate a wide spectrum of optic nerve head characteristics in a large cohort of full-term children aged 4 to 17 years with varying degrees of fetal growth restriction independent of prematurity and fetal overgrowth. The analyses revealed that children and adolescents with severely restricted fetal growth exhibited a significantly thinner pRNFL compared to their average-born counterparts, while children born with moderate fetal growth restriction exhibited a thinner MRW compared to children born AGA. Additionally, vCDR increased with decreasing BW percentile.

In the present study, pRNFL thinning was observed in individuals with severe fetal growth restriction in the primary analysis. Most available data on the impact of growth restriction on pRNFL thickness focus on preterm infants during infancy^14^^,^^15^ and childhood^16^^–^^18^; therefore, the results remain inconclusive with a lack of data on the sole effects of growth restriction independent of prematurity. In two of the few studies, term-born children born with severe fetal growth restriction (aged 5 to 6 years) showed a thinner pRNFL compared to their appropriately sized counterparts,^32^ while another study confirmed this association in term-born individuals born with moderate and severe fetal growth restriction (aged 18 to 52 years).^22^ Our study's primary analysis revealed that pRNFL thinning is already present in children born with severe fetal growth restriction from the age of 4 to 17 years; even after the exclusion of anamnestic history of intraocular pressure, glaucoma, and cerebral bleedings, the effect remained significant. In the Gutenberg Prematurity Study in adults, descriptive analyses described that the reduction in the pRNFL in individuals with fetal growth restriction was mainly present in the nasal region,^22^ while in the present study in children, it was mainly reduced in the superotemporal and inferotemporal regions. One potential explanation is that the regional patterns of pRNFL differences associated with fetal growth restriction vary across different stages of life due to ongoing growth, maturation, refractive development, and thus tilting of the optic nerve head or age-related changes in the retinal nerve fiber layer. In a longitudinal study in adolescents, for example, the nasal pRNFL decreased over time with progressive myopia.^33^ However, the pRNFL thickness in the individuals with extreme fetal growth restriction is the lowest in all sectors in both of our studies; therefore, random variation could also play a role. Surprisingly, in children born with moderate fetal growth restriction, the estimator was positive, although not significant. This suggests the presence of additional underlying factors unaccounted for in our analysis, which may either negatively impact pRNFL thickness in children born with extreme fetal growth restriction or provide protective effects in children born with moderate fetal growth restriction. While our findings highlight regional differences, larger, longitudinal studies are needed to determine whether there is a consistent pattern of pRNFL thinning associated with fetal growth restriction across the life span.

We found a negative association between birth weight percentile and median vCDR. In a previous multicenter study of 2353 12-year-old children born full-term and preterm, the authors found that for each additional kilogram of birth weight, the mean cup-to-disc ratio decreased by 0.0136 (*P* = 0.002). The researchers suggested that this reduction might be due to disrupted maturation of ganglion cells, leading to decreased neuroretinal rim tissue and a larger cup-to-disc ratio.^34^ Furthermore, Fledelius^35^ noted that preterm infants weighing less than 2000 g have a significantly higher cup-to-disc ratio. While some studies have explored the link between birth weight and vCDR, they did not adequately account for prematurity. Overall, our data show that in children aged 4 to 17 years, a decrease of 10 BW percentiles is associated with a 0.01 increase in the median vCDR. While this change may not be clinically relevant for a 10-percentile reduction, the transition from being born AGA (median vCDR = 0.26 in our cohort) to being born with moderate fetal growth restriction corresponded to an approximate 15% increase in the median vCDR in our participants, which may predispose these children to a lower reserve capacity regarding glaucomatous changes in later life.

There are little data on the association between restricted fetal growth and BMO in individuals born at term. In their analysis, Lee et al.^36^ found no descriptive differences between their groups (which included both preterm infants without retinopathy of prematurity [ROP] and those treated for ROP), but interestingly, they observed a negative correlation between birth weight and BMO diameter in their preterm group. Our data indicate that there are fewer effects of altered fetal growth on the BMO.

Lee et al.^36^ reported an inverse relationship between MRW and both gestational age and birth weight in children born preterm. However, direct comparisons with our study are difficult, as their analysis did not exclude preterm individuals, and according to our data, MRW was not affected by severely restricted fetal growth but only moderately restricted fetal growth. Although this association remained significant in the sensitivity analysis in the cohort without an anamnestic history of intraocular pressure, glaucoma, and cerebral bleedings, the mere association with moderate fetal growth restriction indicated that there may be additional confounding factors that we could not test for. Further studies could therefore focus on whether factors in children born with moderate fetal growth restriction explain the isolated decreasing effect on the MRW in this group or potentially find protective factors in the treatment of children born with severe fetal growth restriction that may decrease the risk of a decrease in MRW (e.g., increased length of breastfeeding in this group).

Regarding fetal overgrowth, while pRNFL was descriptively higher in children born with moderate fetal overgrowth (*P* ≤ 0.05) we did not find any significant associations with pRNFL thickness, MRW, or BMO and fetal overgrowth. vCDR was descriptively smaller in the children born with fetal overgrowth, and the quantile regression showed a significant association of birth weight percentile and median vCDR. This indicates that there are no consistent influences of fetal overgrowth on the optic nerve head parameters pRNFL, MRW, and BMO at an age of 4 to 17 years, while there seems to be an effect on vCDR.

### Clinical Consequences of Adverse Fetal Growth

The current findings hold significant clinical implications considering that 10% of newborns worldwide experience fetal growth restriction. Our findings highlight the importance of monitoring ocular health in these individuals. As it is still unknown if the reduction in observed optic nerve head parameters such as pRNFL, MRW, or vCDR in individuals with fetal growth restriction represents a normal variant or if the changes indeed pose an increased risk for glaucoma or a higher predisposition for it, clinicians need to be aware of the diagnostic irregularities in these parameters and keep the potential predisposition for glaucoma in mind. Some authors speculate that these associations result from retrograde trans-synaptic degeneration, particularly in individuals who experienced perinatal complications,^37^ while others proposed that early growth restriction and neurologic lesions during neurodevelopment disrupt retinal structure formation and adaptive mechanisms.^22^ This may be of particular importance because pRNFL thinning correlates with lower brain volume^8^ and may be accompanied by a reduced reserve capacity for degenerative diseases such as glaucoma.^22^

### Strengths and Limitations

This study has certain limitations. It was conducted at a single hospital, which may limit the generalizability of the findings. Additionally, some potential participants could not be contacted or declined to take part, which might affect the representativeness of the sample. Since most participants were of white ethnicity, the results are most applicable to this group. Furthermore, as an exploratory study, multiple testing adjustments were not made, which should be considered when generalizing the results.

Despite these limitations, the study has notable strengths. It includes a large sample size and, to the best of our knowledge, is the first to focus specifically on several optic nerve characteristics in individuals born full-term with altered fetal growth in childhood and adolescence. The detailed perinatal data from medical records enabled a comprehensive assessment of relevant factors, and all measurements were conducted using standardized protocols to reduce variability.

## Conclusions

Based on OCT imaging data of the pRNFL in this study, severe fetal growth restriction appears to affect the optic nerve head in term-born children and adolescents, suggesting a possible reduction in neuronal reserve. This reduction may indicate a previously abnormal neurodevelopment in these children.

## Supplementary Material

Supplement 1
