# Supplementary material for: Fetal Growth Restriction Is Associated With Altered Optic Nerve Head Morphology in Term-Born Children and Adolescents
Source: Invest Ophthalmol Vis Sci. 2025 Apr 15;66(4):35. doi: 10.1167/iovs.66.4.35 (PMC12007673; doi:10.1167/iovs.66.4.35)
Supplement: Supplement 1 [file iovs-66-4-35_s001.pdf]

Supplemental Material:

Figure S1: Flow Chart of the recruiting algorithm for this study

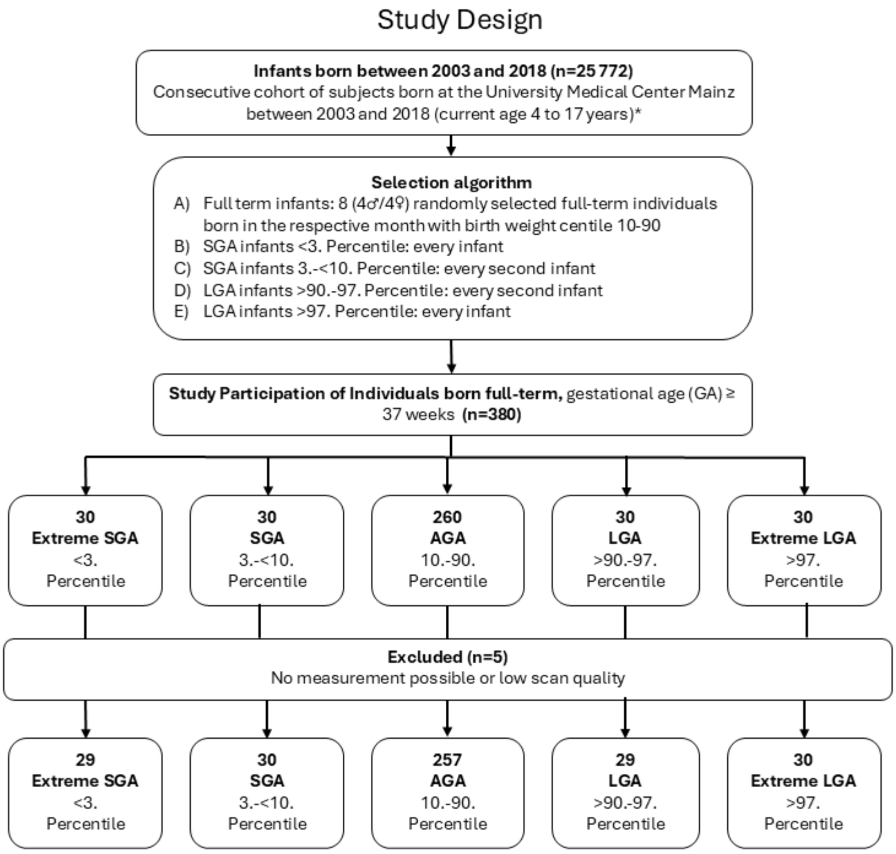

Legend: GA – gestational age; SGA – small for gestational age (fetal growth restriction); LGA – large for gestational age (fetal overgrowth)

Figure S2: Image of an Overview of the Measurements of the Bruch's-Membrane-Opening (central image) and the Minimum rim width (surrounding images with arrowed MRW measuremens)

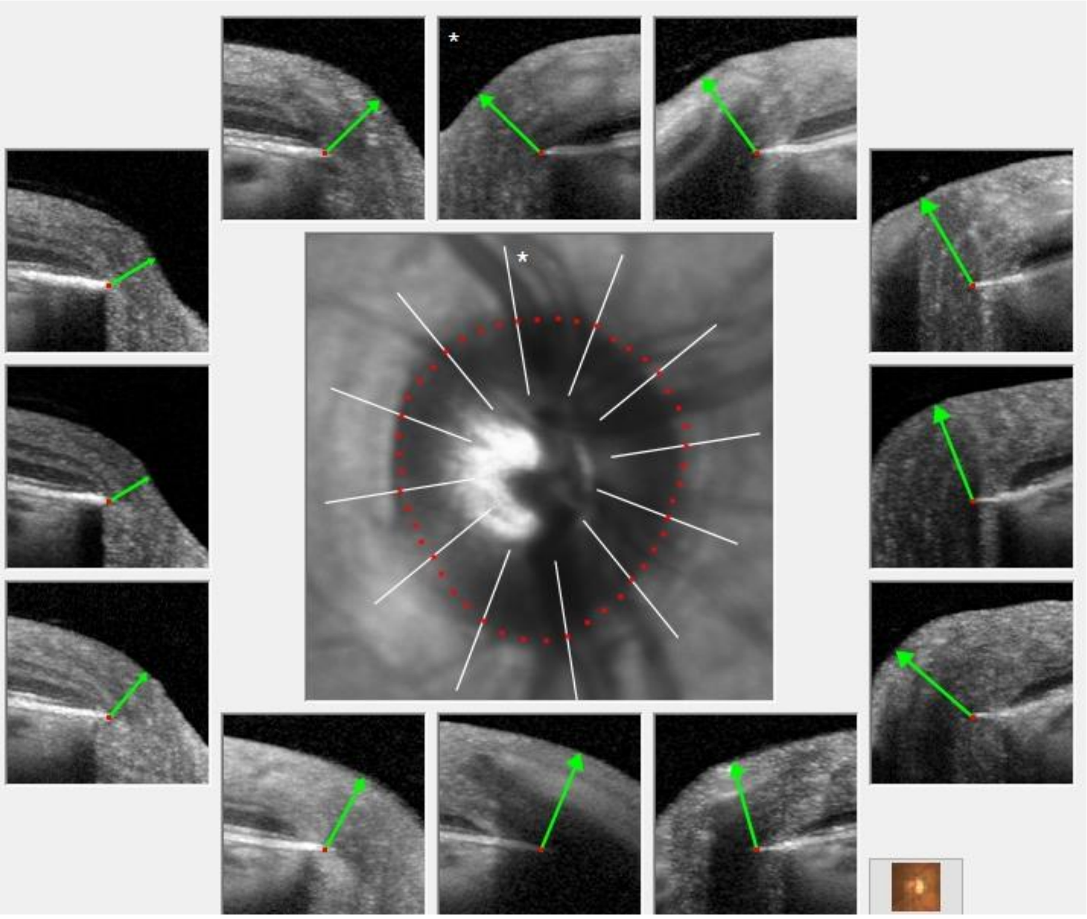

Figure S3: OCT Image of the measured RNFL

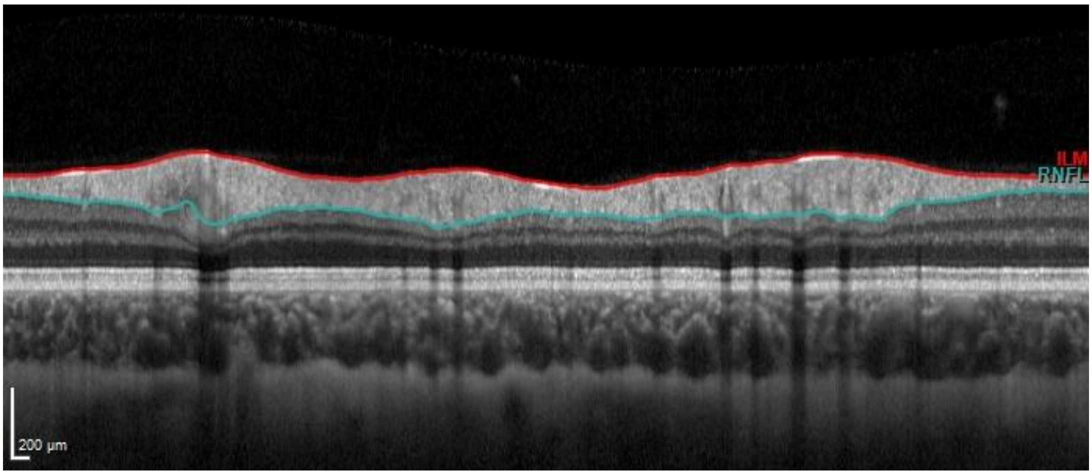

Supplemental Table 1

**Factor**	**multivariable**	**p**	**multivariable**	**p**	**multivariable**	**p**
Sensitivity Analysis without anamnestic history of high IOP, glaucoma, cerebral bleeding, cerebral ischemia, neurodegenerative diseases and cerebral paresis						
	Excluded: Anamnestic History of Glaucoma		Excluded: Anamnestic History of high IOP and Glaucoma		Excluded: Anamnestic History of high IOP and Glaucoma, cerebral bleeding, cerebral ischemia, neurodegenerative diseases and cerebral paresis	
**RNFL**	B [95% CI]	p	B [95% CI]	p	B [95% CI]	p
Gestational age	-0.08 [-1.03; 0.87]	0.87	-0.2 [-1.14; 0.75]	0.68	-0.2 [-1.14; 0.75]	0.68
Birth weight percentile						
<3. Percentile	-5.00 [-9.49; -0.51]	**0.03**	-5.31 [-9.79; -0.83]	**0.02**	-5.31 [-9.79; -0.83]	**0.02**
3.-<10. Percentile	1.14 [-3.37; 5.66]	0.62	0.9 [-3.61; 5.4]	0.7	0.9 [-3.61; 5.4]	0.7
10.-<90. Percentile	Ref.		Ref.		Ref.	
90.-<97. Percentile	2.61 [-0.39; 5.61]	0.09	2.49 [-0.52; 5.51]	0.1	2.49 [-0.52; 5.51]	0.1

>97. Percentile	3.06 [-0.39; 6.51]	0.08	2.95 [-0.51; 6.4]	0.09	2.95 [-0.51; 6.4]	0.09
Gestational diabetes (Yes)	-0.08 [-1.03; 0.87]	0.87	0.51 [-3.34; 4.37]	0.79	0.51 [-3.34; 4.37]	0.79
Smoking during pregnancy (Yes)	-1.91 [-6.17; 2.34]	0.38	-2.13 [-6.41; 2.16]	0.33	-2.13 [-6.41; 2.16]	0.33
Breastfeeding (Yes)	1.73 [-1.22; 4.69]	0.25	1.94 [-1.03; 4.9]	0.2	1.94 [-1.03; 4.9]	0.2
**MRW**						
Gestational age	-6.01 [-12.51; 0.49]	0.07	-6.54 [-13.1; 0.02]	0.05	-6.54 [-13.1; 0.02]	0.05
Birth weight percentile						
<3. Percentile	-7.61 [-41.21; 25.99]	0.66	-8.57 [-42.18; 25.04]	0.62	-8.57 [-42.18; 25.04]	0.62
3.-<10. Percentile	-32.46 [-51.52; -13.4]	**<0.001**	-33.28 [-52.35; -14.22]	**<0.001**	-33.28 [-52.35; -14.22]	**<0.001**
10.-<90. Percentile	Ref.		Ref.		Ref.	
90.-<97. Percentile	30.56 [-13.19; 74.31]	0.17	30.07 [-13.63; 73.76]	0.18	30.07 [-13.63; 73.76]	0.18
>97. Percentile	18.27 [-20.84; 57.37]	0.36	18 [-21; 56.99]	0.37	18 [-21; 56.99]	0.37
Gestational diabetes (Yes)	-19.04 [-40.86; 2.79]	0.09	-19.8 [-41.66; 2.06]	0.08	-19.8 [-41.66; 2.06]	0.08
Smoking during pregnancy (Yes)	0.28 [-30.51; 31.07]	0.99	-0.24 [-31.06; 30.59]	0.99	-0.24 [-31.06; 30.59]	0.99
Breastfeeding (Yes)	27.12 [8.19; 46.06]	**<0.001**	27.87 [8.88; 46.87]	**<0.001**	27.87 [8.88; 46.87]	**<0.001**
